# Cigarette Smoking, miR-27b Downregulation, and Peripheral Artery Disease: Insights into the Mechanisms of Smoking Toxicity

**DOI:** 10.3390/jcm10040890

**Published:** 2021-02-22

**Authors:** Tiago Pereira-da-Silva, Patrícia Napoleão, Marina C. Costa, André F. Gabriel, Mafalda Selas, Filipa Silva, Francisco J. Enguita, Rui Cruz Ferreira, Miguel Mota Carmo

**Affiliations:** 1 Department of Cardiology, Hospital de Santa Marta, Centro Hospitalar Universitário de Lisboa Central, 1169-024 Lisbon, Portugal; mafalda.selas@gmail.com (M.S.); felipafernandes@gmail.com (F.S.); cruzferreira@netcabo.pt (R.C.F.); 2 NOVA Doctoral School, NOVA Medical School|Faculdade de Ciências Médicas, Universidade NOVA de Lisboa, 1169-056 Lisbon, Portugal; 3 Instituto de Medicina Molecular João Lobo Antunes, Faculdade de Medicina, Universidade de Lisboa, 1649-028 Lisbon, Portugal; napoleao.patricia@gmail.com (P.N.); marinacosta@medicina.ulisboa.pt (M.C.C.); andre.gabriel@medicina.ulisboa.pt (A.F.G.); fenguita@medicina.ulisboa.pt (F.J.E.); 4 Cardiomics Unit, Centro Cardiovascular da Universidade de Lisboa, Faculdade de Medicina, Universidade de Lisboa, 1649-028 Lisbon, Portugal; 5 Chronic Diseases Research Center (CEDOC), NOVA Medical School, Faculdade de Ciências Médicas, Universidade NOVA de Lisboa, 1150-082 Lisbon, Portugal; mabmc@sapo.pt

**Keywords:** atherosclerosis, cigarette smoking, miR-27b, peripheral artery disease

## Abstract

Cigarette smoking is a risk factor for the development of peripheral artery disease (PAD), although the proatherosclerotic mediators of cigarette smoking are not entirely known. We explored whether circulating microRNAs (miRNAs) are dysregulated in cigarette smokers and associated with the presence of PAD. Ninety-four participants were recruited, including 58 individuals without and 36 with PAD, 51 never smokers, 28 prior smokers, and 15 active smokers. The relative expression of six circulating miRNAs with distinct biological roles (miR-21, miR-27b, miR-29a, miR-126, miR-146, and miR-218) was assessed. Cigarette smoking was associated with the presence of PAD in multivariate analysis. Active smokers, but not prior smokers, presented miR-27b downregulation and higher leukocyte, neutrophil, and lymphocyte counts; miR-27b expression levels were independently associated with active smoking. Considering the metabolic and/or inflammatory abnormalities induced by cigarette smoking, miR-27b was independently associated with the presence of PAD and downregulated in patients with more extensive PAD. In conclusion, the atheroprotective miR-27b was downregulated in active smokers, but not in prior smokers, and miR-27b expression was independently associated with the presence of PAD. These unreported data suggest that the proatherogenic properties of cigarette smoking are mediated by a downregulation of miR-27b, which may be attenuated by smoking cessation.

## 1. Introduction

Cigarette smoking is a major health hazard, being accountable for substantial cardiovascular morbidity and mortality worldwide due to its proatherogenic effects [1]. Cigarette smoking increases the risk of atherosclerosis development by several fold and is a more influential risk factor for peripheral artery disease (PAD) than for atherosclerosis of other territories, including coronary arteries [2]. Some of the mechanisms associated with cigarette-smoking-induced atherogenesis include the activation of inflammation, dysregulation of the lipid metabolism, increase in oxidative stress, and endothelial dysfunction [1,2]. Nevertheless, the pathophysiology associated with the initiation and progression of atherosclerosis secondary to cigarette smoking is not entirely known [1,2].

MicroRNAs (miRNAs) are small noncoding RNA molecules that regulate the genetic expression at the post-transcriptional level [3]. Cigarette smoking is associated with an altered expression of circulating miRNAs, including an upregulation of pro-inflammatory miRNAs [4,5]. On the other hand, specific miRNAs participate in different steps of atherogenesis, and a dysregulated expression of circulating miRNAs was described in patients with stable atherosclerosis of different territories [3,6]. However, to the best of our knowledge, coexistent associations among cigarette smoking, miRNA dysregulation, and the presence of atherosclerosis in humans have not been reported. The identification of miRNA dysregulation in association with both cigarette smoking and atherosclerosis could provide insights into the pathophysiology of cigarette-smoking toxicity. In fact, miRNAs may mediate the causal relationship between cigarette smoking and atherosclerosis development, especially PAD, which is particularly influenced by cigarette smoking [2].

Of the diversity of miRNAs associated with atherosclerosis regulation, miR-27b, miR-21, miR-29a, miR-126, miR-146, and miR-218 participate in distinct pathways and/or have distinct mechanisms of action, as described in experimental models, and were also reported to be dysregulated in patients with stable atherosclerosis [3,6,7,8,9]. Of note, miR-27b is a pleiotropic miRNA, and its expression is associated not only with atherosclerotic disease but also with non-atherosclerotic cardiovascular processes, such as cardiomyocyte hypertrophy and non-cardiovascular diseases, including cancer, non-alcoholic fatty liver disease, and viral infections [10,11,12,13,14,15,16,17,18]. Specifically, regarding atherosclerosis, miR-27b regulates lipid metabolism, development of lipid-induced atherosclerotic lesions, vascular inflammation, endothelial function, and angiogenesis [19,20,21,22,23,24,25,26]. Overall, the reported effects of miR-27b in experimental models are atheroprotective [19,20,21,22,23,24,25,26]. Among the relevant roles of the remaining miRNAs in the development and expression of atherosclerosis, miR-21 regulates vascular smooth cell and endothelial cell functions, miR-29a regulates fibrosis and extracellular matrix composition, miR-126 regulates endothelial function in response to shear stress, miR-146 regulates endothelial function in response to inflammatory cytokines, and miR-218 regulates endothelial cell migration [3,7,8].

We explored whether circulating miRNAs are simultaneously dysregulated in cigarette smokers and associated with the presence of PAD. 

## 2. Materials and Methods

The study protocol was approved by the ethics committees of the involved institutions (Centro Hospitalar Universitário de Lisboa Central, Nr. 245/2015, in 2015, and the NOVA Medical School | Faculdade de Ciências Médicas, Universidade NOVA de Lisboa, Nr. 000176, in 2015). The investigation conformed to the principles outlined in the Helsinki Declaration. All the participants signed informed consent forms.

### 2.1. Recruitment of Participants

Two groups of participants from our center were recruited, with and without PAD. PAD was defined as a significant (≥50%) stenosis on Doppler ultrasound at rest [27,28] or the combination of chronic claudication and an ankle-brachial index equal to or less than 0.9 [28,29]. Doppler ultrasound was performed for the characterization of PAD, according to a standardized protocol, using the GE Logiq S7 Expert Ultrasound System, and measurements were performed while following published guidelines [28,29].

We excluded patients with critical limb ischemia (with ischemic rest pain), those with acute ischemic events within 12 months in any arterial territory, those with lower-extremity bypass surgery performed within 12 months, those with any prior percutaneous arterial treatment, those with heart failure, hemodynamically significant valvular heart disease, hematological disorders, active infection (based on symptoms and/or signs, including fever, and leukocyte count, white blood cell differential, and C-reactive protein levels), history of malignancy, chronic kidney disease (stage 4 or 5), or severe hepatic dysfunction, those under 18 years of age, and those unable or unwilling to consent to study participation.

### 2.2. Data Collection

Data were collected prospectively after patient inclusion. A standardized record including clinical, demographic, laboratory, echocardiographic, and Doppler ultrasound data was obtained from each participant. Participants were classified according to cigarette-smoking status as never smokers, prior smokers (if cessation occurred at least six months before), or active smokers (in cases of daily cigarette smoking, irrespective of the number of cigarettes) [30].

### 2.3. Candidate miRNAs

Six candidate miRNAs (miR-21, miR-27b, miR-29a, miR-126, miR-146, and miR-218) were selected based on the following criteria: miRNAs are associated with the regulation of atherosclerosis development and expression in experimental models [3,7,8]; each of the miRNAs regulates distinct pathways of atherosclerotic disease and/or has distinct mechanisms of action [3,7,8]; miRNAs were reported to be dysregulated in patients with stable atherosclerosis [6,9].

### 2.4. Quantification of Expression Levels of Candidate miRNAs

Peripheral blood was collected early in the morning under fasting conditions. Serum was separated by centrifugation (500× *g* for 10 min) within 15 min of sampling. Aliquots were stored at −80 °C, and samples were thawed only once.

Total RNA was extracted from serum samples using the miRCURY™ RNA Isolation Kit (Qiagen, Hilden, Germany). Complementary DNA was synthesized from total RNA using the Universal complementary DNA (cDNA) synthesis kit from the miRCURY™ LNA miRNA system (Qiagen, Hilden, Germany). miRNA amplification was performed using quantitative reverse-transcription polymerase chain reaction (using the miRCURY™ LNA SYBR Green PCR Kit and LNA™ PCR primers, Qiagen, Hilden, Germany), and the melting curve was determined according to the following conditions: 95 °C for 10 min, followed by 45 cycles of 95 °C for 10 s and 60 °C for 60 s. All reactions were performed in triplicate. The amplification data were assessed using DataAssist™ Software v3.01. Cycle threshold (Ct) values greater than 40 were considered undetermined [31,32,33,34]. The relative expression levels of the six candidate miRNAs were calculated using the delta Ct (ΔCt) method, normalizing for the UniSp6 RNA spike-in control [34,35,36,37]. Higher ΔCt miRs represent lower circulating levels of the candidate miRNAs [34,35,36,37]. Where appropriate, the fold-change in the miRNA expression levels was expressed using the Livak method (2^−ΔΔCt^) [38].

### 2.5. Statistical Analysis

Discrete variables are presented as frequencies (percentages); continuous variables are presented as the mean (standard deviation) in normally distributed data or median (interquartile range (IQR)) in variables without a normal distribution (Shapiro–Wilk test). Categorical variables were analyzed using the chi-square or Fisher’s exact tests. Continuous variables were analyzed using Student’s *t*-test or the Mann–Whitney test when normality was not verified. Comparisons between multiple groups were performed using an analysis of variance (ANOVA) in normally distributed data and the Kruskal–Wallis test in variables without a normal distribution; the Bonferroni post-hoc correction was used for multiple pairwise comparisons. Pearson’s correlation was used to test correlations between continuous variables. Three distinct multivariable logistic regression models were successively tested: (1) using classical cardiovascular risk factors as the independent variables and PAD as the dependent variable; (2) using metabolic and inflammatory data and miRNA expression levels as the independent variables and active smoking as the dependent variable; and (3) using miRNAs and other laboratory parameters dysregulated in cigarette smokers as the independent variables and PAD as the dependent variable. Variables with a *p*-value of <0.10 in univariate analyses were tested in the multivariable models. A correction for collinearity was performed as appropriate. The level of significance was set at α = 0.05. Analyses were conducted using SPSS software, version 26.0 (IBM Corp, Armonk, NY, USA).

## 3. Results

### 3.1. Characteristics of Participants According to the Presence of PAD and Cigarette-Smoking Status

A total of 94 participants were recruited, including 58 without and 36 with PAD (Table 1). Patients with PAD presented a higher prevalence of classical cardiovascular risk factors (including cigarette smoking), concomitant coronary and carotid artery disease, and use of antiplatelet and statin therapy, as well as higher creatinine levels, compared to patients without PAD. The ΔCt miR-27b and ΔCt miR-146 values were significantly higher in patients with PAD (Table 1), corresponding to a 17.0- and 3.4-fold downregulation of miR-27b and miR-146 [38], respectively, in patients with PAD.

Of the 94 participants, 51 were never smokers, 28 were prior smokers, and 15 were active smokers (Table 2). The mean pack-year was 53 (22) in prior smokers and 45 (21) in active smokers, with a median time from cigarette-smoking cessation of 10 (7–11) years in prior smokers. The prevalence of PAD and the proportion of bilateral PAD (among patients with PAD) increased from never smokers to prior smokers and to active smokers. Moreover, the leukocyte, neutrophil, and lymphocyte counts were higher in active smokers compared with other groups. MiR-27b was the only dysregulated miRNA according to cigarette-smoking status; the ΔCt miR-27b values were significantly higher in active smokers compared with prior smokers (*p* = 0.004; corresponding to a downregulation of miR-27b in active smokers), and there was a trend for higher ΔCt miR-27b values in active smokers compared with never smokers (*p* = 0.053; corresponding to a trend for downregulation of miR-27b in active smokers), with no significant differences between never smokers and prior smokers.

**Figure 1:**
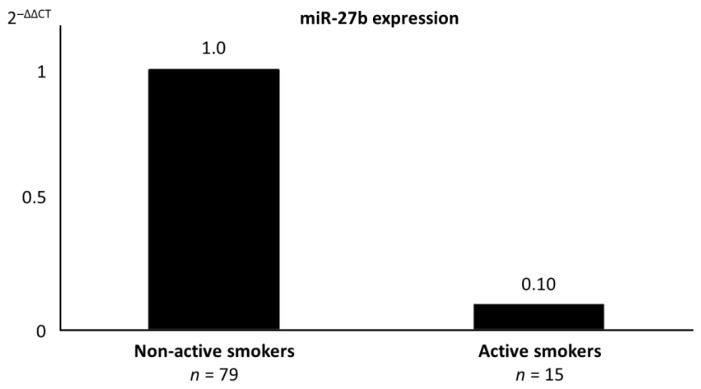
Relative expression of miR-27b according to the cigarette-smoking status. The relative expression of miR-27b (2^−ΔΔCt^) is presented for non-active smokers, including never smokers and prior smokers, and for active smokers.

Active smokers presented significantly higher ΔCt miR-27b values (22.00 (4.35)) compared with non-active smokers, including never smokers and prior smokers (18.66 (4.33)), corresponding to a 10.0-fold downregulation of miR-27b expression levels in active smokers [38] (Figure 1).

### 3.2. Cigarette Smoking Was Independently Associated with the Presence of PAD

Considering all the risk factors for the development of cardiovascular disease that differed according to the presence of PAD in the univariate analysis, the age, cigarette-smoking status, and creatinine levels were independently associated with the presence of PAD in the multivariate logistic regression analysis (Table 3).

### 3.3. MiR-27b Was Dysregulated in Active Smokers Independently of Other Metabolic and Inflammatory Parameters

Considering the metabolic and inflammatory parameters and the miRNAs dysregulated in active smokers in the univariate analysis, the leukocyte count and ΔCt miR-27b were independently associated with active smoking in the multivariate logistic regression analysis (Table 4).

### 3.4. MiR-27b Was Independently Associated with the Presence of PAD

Considering all the metabolic and inflammatory parameters and the miRNA (miR-27b) dysregulated in active smokers in the univariate analysis, only ΔCt miR-27b was independently associated with the presence of PAD (β = 1.13, 95% confidence interval: 1.01–1.28, *p* = 0.037). Moreover, ΔCt miR-27b was significantly higher in bilateral PAD (24.0 (17.2–25.7)) compared with unilateral PAD (20.3 (15.1–23.2), *p* = 0.041 vs. bilateral PAD) and with absent PAD (18.3 (14.8–21.6), *p* = 0.004 vs. bilateral PAD). Such results corresponded to a 4.0- and 52.0-fold reduction in miR-27b in bilateral PAD compared with unilateral and absent PAD, respectively [38].

## 4. Discussion

In this prospective study, three main findings stood out: cigarette smoking was associated with the presence of PAD, cigarette smoking was associated with miR-27b downregulation, and miR-27b downregulation was associated with the presence and severity of PAD. These results suggest that miR-27b mediates the proatherogenic effects of cigarette smoking.

Cigarette smoking is a recognized causal risk factor for PAD development [1,2]. On the other hand, the expression of circulating miRNAs is influenced by exogenous factors, such as cigarette smoking [4]. In this study, the atheroprotective miR-27b was downregulated in active smokers compared with non-active smokers (including never smokers and prior smokers), independently of other metabolic and inflammatory parameters. These results suggest a detrimental effect of active cigarette smoking on miR-27b expression, similar to the reported effect of cigarette smoking on other atheroprotective miRNAs [39]. MiR-27b was significantly downregulated in active smokers compared with prior smokers but not with never smokers, although there was a trend towards miR-27b downregulation in active smokers compared with never smokers. The latter may be explained by the limited sample size used in this study. Based on an extensive post-hoc analysis, the absence of differences in miR-27b expression levels between active smokers and never smokers is difficult to explain, and we did not find additional factors that could contribute to such results. Nevertheless, the numerical differences between active smokers and never smokers were close to the margin of statistical significance and are consistent with a culprit effect of active smoking in downregulating miR-27b compared with prior smokers and, likely, never smokers as well. There were no significant differences in miR-27b expression levels between prior smokers and never smokers. Such results may be explained by a sensitivity of miR-27b expression levels to active smoking without a legacy effect [39,40]. Specifically, smoking cessation was reported to completely revert the dysregulation of some miRNAs observed in active smokers [39,40], and this may also be the case of miR-27b. These data reinforce the atheroprotective effects of smoking cessation. Published data addressing the effect of smoking on miR-27a/b expression are limited [39,40,41]. Of two studies addressing miR-27a, one reported an upregulation and the other reported no dysregulation in active smokers [39,40]. Nevertheless, miR-27a and miR-27b may differ in their mechanisms of action and expression levels in specific diseases, and the aforementioned results concerning miR-27a expression in active smokers may not be applicable to miR-27b [42,43,44]. Specifically, for miR-27b, a nonsignificant downregulation was reported in human oral keratinocytes in association with cigarette smoke exposure [41]. In our study, the downregulation of circulating miR-27b in active smokers was significant after adjusting for other metabolic and inflammatory parameters.

The dysregulation of some circulating miRNAs is known to be associated with the development of atherosclerosis, and miR-27b was reported to be atheroprotective on the basis of experimental studies [19,20,21,22,23,24,25,26]. This possibly explains the association between the reduced miR-27b expression and the higher prevalence and severity of PAD observed in this study. Complementary atheroprotective mechanisms of miR-27b were previously reported [19,20,21,22,23,24,25,26]. Xie W. et al. [19] elegantly showed that miR-27 reduces vascular lipid accumulation, partially mediated by the suppression of expression of scavenger receptors associated with lipid uptake in vascular macrophages. Interestingly, systemic treatment with miR-27 decreased aortic plaque size and lipid content in mice [19]. Consistently, in another study, miR-27b was identified as a crucial regulatory hub in lipid metabolism in human and mouse liver and was shown to downregulate the expression of key genes involved in lipid metabolism, including Angptl3 and Gpam, which mitigates the accumulation of lipids in circulation [20]. Regarding inflammation, miR-27b downregulates lipoprotein lipase gene expression and thereby reduces vascular inflammatory response, which limits atherogenesis [19]. Moreover, miR-27b restrains the activity of NF-κB and the production of several pro-inflammatory factors, including interleukin (IL)-1β, IL-6, and tumor necrosis factor alpha [21], inhibits interleukin-17-induced monocyte chemoattractant protein-1 [22] and targets Bcl-2-associated athanogene 2 in macrophages [21]. This contributes to a decreased monocyte-macrophage activation [23]. Considering the key role of vascular inflammation, particularly the monocyte-macrophage activation, in the development and expression of atherosclerosis, the aforementioned molecular mechanisms are consistent with the atheroprotective effects of miR-27b [45,46]. On the other hand, as miR-27b represses repulsive semaphorins, especially semaphorin 6A, it facilitates the formation of tight endothelial monolayers and stable vessels in response to shear stress [24]. In addition, miR-27b was identified as a proangiogenic miRNA [25], regulating angiogenesis through the angiogenic inhibitor semaphorin 6A and Notch ligand Dll4 [23,26]. Regarding studies addressing miR-27b expression in patients with PAD, data is conflicting. Signorelli et al. [11] reported an upregulation of miR-27b in patients with PAD compared with controls. On the other hand, Stather et al. [10] reported a downregulation of miR-27b in patients with PAD, and our findings are consistent with their results. Contrary to the study by Signorelli et al. [11], Stather et al. [10] confirmed the presence of PAD using imaging methods, used one derivation and two validation sample sets, reported the diagnostic accuracy of miR-27b for detecting PAD, and presented a pathway enrichment analysis. There is a known biological variation in miRNAs measurements, occasionally with conflicting expression levels among different studies focused on the same disease [47]. The miR-27b downregulation observed in our study reinforces the results of the study by Stather et al. [10], where a very robust methodology was used.

**Figure 2:**
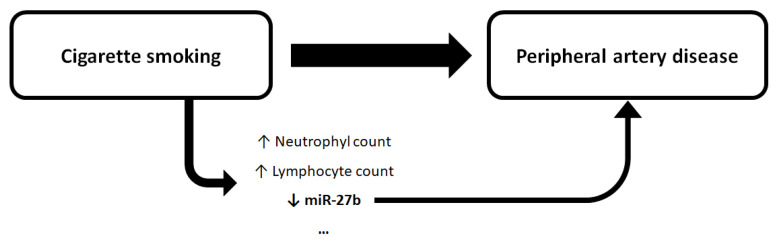
Putative role of miR-27b in the toxicity induced by cigarette smoking. Cigarette smoking was associated with the presence of peripheral artery disease and with miR-27b downregulation; downregulation of the atheroprotective miR-27b was associated with the presence and severity of peripheral artery disease; miR-27b may mediate the atherogenesis induced by cigarette smoking. The suspension points represent metabolic and inflammatory parameters potentially dysregulated in association with cigarette smoking, in addition to neutrophil count, lymphocyte count, and miR-27b expression.

The downregulation of miR-27b in active smokers and the independent association between miR-27b and the presence of PAD in this study suggest that miR-27b is downregulated due to active cigarette smoking and that such dysregulation contributes to PAD (Figure 2). The results are, therefore, consistent with miR-27b acting as a mediator of cigarette-smoking toxicity, specifically in PAD development.

In this study, miR-146 was also downregulated in patients with PAD. MiR-146 is induced in endothelial cells in response to pro-inflammatory cytokines and acts as a negative feedback regulator of inflammatory signaling in endothelial cells by dampening the activation of pro-inflammatory transcriptional programs, including the NF-κB, AP-1, and MAPK/EGR pathways, and by promoting eNOS expression [3,7,48]. Moreover, miR-146a was reported to decrease endothelial inflammation by inhibiting NAPDH Oxidase 4 expression in a diabetic atherothrombosis model [49]. The anti-inflammatory effects of miR-146 contribute to counteracting atherogenesis [3,7,48,49] and may explain the association between miR-146 downregulation and the presence of PAD observed in our study. The remaining miRNAs (miR-21, miR-29a, miR-126, and miR-218) were not dysregulated in association with PAD, despite their involvement in biological pathways associated with atherosclerosis regulation, as reported in experimental studies [48,50,51,52,53,54,55,56,57,58,59,60]. A comprehensive review of their biological roles is out of the scope of this study, but some of the main targets are herein addressed. MiR-21 has both atheroprotective and proatherogenic effects [50]. It enhances vascular smooth muscle cell migration and proliferation by targeting TSP-1 and c-Sk [50,51]. On the other hand, miR-21 inhibits both endothelial cell apoptosis, through PTEN [52], and endothelial cell proliferation, through RhoB [53]. In addition, miR-21 promotes inflammatory activation of endothelial cells by targeting PPARα, which induces the expression of adhesion molecules and cytokines [54]. MiR-29a acts in distinct pathways, as it represses transcripts of several components of the extracellular matrix; of note, miR-29a downregulates the expression of ELN, COL1A1, and COL3A1, resulting in a reduction of the elastin and collagen content in the atherosclerotic plaque [55,56]. In fact, antagonizing the antifibrotic effect of miR-29a leads to a reduced size of atherosclerotic lesions, enhanced fibrous cap thickness, and reduced necrotic zones [55]. MiR-126 promotes endothelial proliferation and limits atherosclerosis by suppressing Dlk1 [57]. Interestingly, miR-126 is a mechanosensitive miRNA that is downregulated through the transcription factor klf2a in response to disturbed flow and shear stress [58]. Finally, miR-218 regulates Slit/Robo signaling through the repression of Robo1, Robo2, and glucuronyl C5-epimerase and thereby regulates endothelial cell migration and vascular patterning [59,60]. As supported by the aforementioned experimental data, further clinical studies are warranted to confirm the role of these miRNAs as biomarkers of atherosclerosis. 

There are strengths of this study that should be acknowledged. As far as we know, we describe for the first time coexistent associations among cigarette-smoking status, dysregulation of circulating miR-27b, and PAD. The results were consistent with the aforementioned biological roles of miR-27b in experimental studies [19,20,21,22,23,24,25,26]. Moreover, the miR-27b downregulation in more severe, bilateral PAD further added to the consistency of the results. Importantly, the multivariable analyses carried out in this study, adjusting for confounders, contributed to the accuracy of our findings.

Our study has some limitations. The results indicate associations among cigarette smoking, miR-27b dysregulation, and PAD development, but not a causal effect. Nevertheless, the adjustment for confounders in the multivariable analyses and the consistency of the results with the aforementioned experimental data [19,20,21,22,23,24,25,26] suggest that miR-27b is very likely a mediator in such a pathophysiology chain. Of note, this is a single-center study that included only European participants, which may limit the applicability of results to other clinical settings. Populations with different ethnicities may express distinct microRNA profiles, either in healthy individuals or in specific disease subsets [61,62]. Therefore, further multicentric studies recruiting participants from distinct geographical areas are warranted for external validation of our findings.

## 5. Conclusions

Cigarette smoking was associated with the presence of PAD. Active smokers, but not prior smokers, presented a downregulation of miR-27b, and such dysregulation was associated with the presence and severity of PAD. These unreported data suggest that miR-27b mediates the proatherogenic effects of cigarette smoking and that cigarette smoking cessation may be associated with an attenuation of miR-27b dysregulation. Our results provide insights into the pathophysiology of cigarette-smoking toxicity and associated PAD. 

## Figures and Tables

**Table 1:** Characteristics of participants without and with peripheral artery disease.

Characteristics of Participants	Without Peripheral Artery Disease	With Peripheral Artery Disease	*p*-Value
*n*	58	36	
**Clinical characteristics**			
Age, years	61 (53–70)	68 (60–73)	0.009
Male, *n* (%)	51 (88)	33 (92)	0.419
Hypertension, *n* (%)	42 (72)	36 (100)	<0.001
Dyslipidemia, *n* (%)	48 (83)	35 (97)	0.031
Diabetes mellitus, *n* (%)	15 (26)	17 (47)	0.029
Cigarette-smoking status, *n* (%)			0.001
Never smoker	39 (67)	12 (33)	
Prior smoker	15 (26)	13 (36)	
Active smoker	4 (7)	11 (31)	
LVEF > 50%, *n* (%)	58 (100)	36 (100)	–
Antiplatelet therapy, *n* (%)	37 (64)	35 (97)	<0.001
Statin therapy, *n* (%)	42 (72)	32 (91)	0.023
Coronary artery disease, *n* (%)	32 (55)	36 (100)	<0.001
Carotid artery disease, *n* (%)	12 (55)	18 (50)	0.003
**Peripheral artery disease**			
Bilateral disease, *n* (%)	0 (0)	25 (69)	<0.001
Prior bypass surgery, *n* (%)	0 (0)	8 (22)	<0.001
**Laboratory parameters**			
Hemoglobin, g/dL	13.8 (1.5)	13.6 (1.6)	0.586
Leukocyte count, 10^9^/L	7.0 (1.9)	7.7 (1.7)	0.081
Neutrophil count, 10^9^/L	4.0 (1.7)	4.5 (1.4)	0.192
Lymphocyte count, 10^9^/L	1.9 (1.6–2.3)	2.2 (1.6–2.7)	0.100
Neutrophil/lymphocyte ratio	2.2 (1.0)	2.2 (1.0)	0.943
Platelet count, 10^9^/L	223 (58)	227 (44)	0.749
Fasting glycemia, mg/dL	92 (83–104)	90 (82–121)	0.978
Percentage of glycosylated hemoglobin	5.7 (5.4–6.2)	5.9 (5.5–7.4)	0.295
Creatinine, mg/dL	0.85 (0.77–0.97)	0.94 (0.80–1.34)	0.036
Total cholesterol, mg/dL	164 (130–206)	166 (147–205)	0.781
LDL-cholesterol, mg/dL	92 (72–130)	109 (83–135)	0.250
HDL-cholesterol, mg/dL	43 (34–51)	36 (31–43)	0.062
Triglycerides, mg/dL	115 (71–163)	117 (88–178)	0.429
C-reactive protein, mg/L	4.0 (1.9)	3.6 (1.5)	0.529
**miRNAs** ^1^			
miR-21	14.89 (4.51)	15.99 (4.65)	0.282
miR-27b	18.23 (14.58–21.59)	22.32 (16.50–24.13)	0.032
miR-29a	20.42 (3.57)	21.80 (3.12)	0.152
miR-126	16.89 (14.89–22.46)	19.69 (16.28–24.06)	0.060
miR-146	18.70 (3.40)	20.48 (4.23)	0.048
miR-218	22.69 (22.48–23.33)	14.49 (−8.6–23.50)	0.186

Categorical variables are expressed as the frequency (percentage), and continuous variables are expressed as the mean (standard deviation) or median (interquartile range). HDL—high-density lipoprotein; LDL—low-density lipoprotein; LVEF—left-ventricular ejection fraction; miRNA—microRNA. ^1^ Delta cycle threshold (ΔCt) values are presented for each miRNA (higher ΔCt values correspond to lower miRNA expression levels).

**Table 2:** Characteristics of participants according to cigarette-smoking status.

Characteristics of Participants	Never Smokers	Prior Smokers	Active Smokers	*p*-Value
*n*	51	28	15	
**Clinical characteristics**				
Age, years	65 (56–73)	67 (58–71)	59 (53–68)	0.415
Male, *n* (%)	43 (84)	28 (100)	13 (87)	0.090
Hypertension, *n* (%)	36 (71)	28 (100)	14 (93)	0.002
Dyslipidemia, *n* (%)	43 (84)	26 (93)	14 (93)	0.424
Diabetes mellitus, *n* (%)	17 (33)	10 (36)	5 (33)	0.975
LVEF > 50%, *n* (%)	51 (100)	28 (100)	15 (100)	–
Antiplatelet therapy, *n* (%)	36 (71)	24 (86)	12 (80)	0.298
Statin therapy, *n* (%)	37 (73)	24 (86)	13 (93)	0.156
Coronary artery disease, *n* (%)	31 (61)	24 (86)	13 (86)	0.024
Carotid artery disease, *n* (%)	14 (51)	10 (36)	6 (40)	0.576
**Peripheral artery disease**				
Number of patients	12 (24)	13 (46)	11 (73)	0.001
Bilateral disease, *n* (%)	8 (16)	11 (39)	6 (40)	0.002
Prior bypass surgery, *n* (%)	2 (4)	5 (18)	1 (7)	0.101
**Laboratory parameters**				
Hemoglobin, g/dL	13.4 (1.5)	14.1 (1.4)	14.1 (1.4)	0.063
Leukocyte count, 10^9^/L	6.6 (1.5)	7.3 (1.8)	9.2 (1.8)	<0.001 ^1,2^
Neutrophil count, 10^9^/L	3.8 (3.0–4.4)	3.9 (3.2–5.3)	4.6 (3.7–5.8)	<0.001 ^1,2^
Lymphocyte count, 10^9^/L	1.8 (1.5–2.2)	2.1 (1.6–2.4)	2.3 (1.8–3.5)	0.027 ^1^
Neutrophil/lymphocyte ratio	2.0 (1.5–3.0)	2.2 (1.7–2.7)	2.3 (1.9–2.8)	0.568
Platelet count, 10^9^/L	219 (51)	218 (54)	255 (51)	0.058
Fasting glycemia, mg/dL	92 (83–116)	91 (85–112)	89 (72–117)	0.565
Percentage of glycosylated hemoglobin	5.7 (5.3–7.1)	5.8 (5.4–6.2)	5.9 (5.6–6.1)	0.787
Creatinine, mg/dL	0.86 (0.78–1.08)	0.86 (0.76–1.00)	1.01 (0.75–1.71)	0.447
Total cholesterol, mg/dL	178 (51)	154 (43)	172 (48)	0.060
LDL-cholesterol, mg/dL	108 (41)	89 (38)	116 (32)	0.059
HDL-cholesterol, mg/dL	42 (34–51)	41 (29–46)	35 (31–42)	0.236
Triglycerides, mg/dL	116 (79–156)	104 (73–178)	158 (112–243)	0.139
C-reactive protein, mg/L	3.5 (1.6)	3.7 (1.8)	4.0 (1.9)	0.339
**miRNAs** ^3^				
miR-21	15.44 (4.40)	14.55 (4.62)	16.42 (5.09)	0.454
miR-27b	19.29 (4.42)	17.44 (3.98)	22.00 (4.35)	0.014 ^2^
miR-29a	21.01 (3.04)	19.74 (3.86)	23.19 (3.12)	0.059
miR-126	17.42 (15.20–23.98)	16.10 (14.89–19.48)	22.40 (17.65–24.15)	0.075
miR-146	20.03 (15.74–22.21)	17.79 (16.26–20.12)	21.69 (18.23–23.58)	0.150
miR-218	22.31 (15.67–23.02)	22.31 (15.67–23.02)	8.45 (−8.46 –21.50)	0.120

Categorical variables are expressed as the frequency (percentage), and continuous variables are expressed as the mean (standard deviation) or median (interquartile range). HDL—high-density lipoprotein; LDL—low-density lipoprotein; LVEF—left-ventricular ejection fraction; miRNA—microRNA. ^1^ *p* < 0.05, active smokers vs. never smokers; ^2^ *p* < 0.05, active smokers vs. prior smokers; ^3^ delta cycle threshold (ΔCt) values are presented for each miRNA (higher ΔCt values correspond to lower expression levels of candidate miRNAs).

**Table 3:** Predictors of peripheral artery disease in multivariate logistic regression analysis.

Independent Predictors	β	95% CI	*p*-Value
Age, years	1.11	1.04–1.18	0.002
Cigarette smoking	4.11	1.89–8.95	0.031
Creatinine, mg/dL	6.29	1.12–33.49	<0.001

95% CI—95% confidence interval.

**Table 4:** Parameters dysregulated in active smokers in multivariate logistic regression analysis.

Independent Predictors	β	95% CI	*p*-Value
Leukocyte count	2.33	1.37–3.94	0.002
ΔCt miR-27b	1.33	1.02–1.72	0.034

95% CI—95% confidence interval, ΔCt—delta cycle threshold.

## Data Availability

The data presented in this study are available on request from the corresponding author. The data are not publicly available due to personal data protection.
